# Design and Performance Analysis of Compact Printed Ridge Gap Waveguide Phase Shifters for Millimeter-Wave Systems

**DOI:** 10.3390/s24144702

**Published:** 2024-07-20

**Authors:** Moath Alathbah, Mohamed S. El-Gendy, Mahmoud Gadelrab, Mohamed Mamdouh M. Ali

**Affiliations:** 1 Department of Electrical Engineering, College of Engineering, King Saud University, Riyadh 11451, Saudi Arabia; 2 Microstrip Department, Electronics Research Institute, Cairo 11843, Egypt; 3 R&D Department, Scientific Microwave Corporation, Montreal, QC H4S 1C1, Canada; 4 Department of Electrical Engineering, Faculty of Engineering, Assiut University, Assiut 71516, Egypt

**Keywords:** cutoff frequency, effective permittivity, phase shifter, printed ridge gap waveguide (PRGW), propagation constant

## Abstract

This paper introduces compact Printed Ridge Gap Waveguide (PRGW) phase shifters tailored for millimeter-wave applications, with a focus on achieving wide operating bandwidth, and improved matching and phase balance compared to single-layer technology. This study proposes a unique approach to achieve the required phase shift in PRGW technology, which has not been previously explored. This study also introduces a novel analytical approach to calculate the cutoff frequency and propagation constant of the PRGW structure, a method not previously addressed. Furthermore, the utilization of multi-layer PRGW technology enables the realization of multi-layer beamforming networks without crossing, thereby supporting wideband operation in a compact size. The proposed design procedure enables the realization of various phase shift values ranging from $0^{\circ }$ to $135^{\circ }$ over a broad frequency bandwidth centered at 30 GHz. A 45-degree phase shifter is fabricated and tested, demonstrating a 10 GHz bandwidth (approximately 33% fractional bandwidth) from 25 GHz to 35 GHz. Throughout the operating bandwidth, the phase balance remains within 45 ± $5^{\circ }$, with a deep matching level of −20 dB. The proposed phase shifter exhibits desirable characteristics, such as compactness, low loss, and low dispersion, making it a suitable choice for millimeter-wave applications, including beyond 5G (B5G) and 6G wireless communications.

## 1. Introduction

The rapid growth of millimeter-wave applications, driven by the increasing demand for high-speed wireless communication systems, such as fifth-generation (5G) networks, has sparked significant interest in the development of compact and efficient phase shifters. Phase shifters play a critical role in beamforming, antenna steering, and signal processing, enabling enhanced data rates, improved coverage, and increased network capacity. While 5G continues to revolutionize the way in which we interact with the world, the evolution to 6G is already in the early applied research phase, emphasizing the need for advanced millimeter-wave technologies [1,2,3]. In this context, a substantial body of research has been dedicated to exploring novel techniques and technologies for achieving desirable phase shifter characteristics, particularly for applications such as beamforming networks, including Butler matrices. Compactness is crucial for realizing the full potential of phase shifters in these networks, as it enables the integration of multiple components in a limited space, facilitating the advancement of 6G technical possibilities and trends [4,5,6].

The current research landscape exposes a significant gap in advancing phase shifter technology using Printed Ridge Gap Waveguides (PRGWs) for millimeter-wave frequencies. While there has been some progress, it is worth noting that only one study [7] has managed to utilize Schiffman phase shifters effectively to construct a linear Butler matrix and implement a 1D beam scanning antenna array. However, this approach faced limitations due to its narrow operational bandwidth, restricted to 20%, and large size. The Schiffman technique, although effective, encounters challenges at mmWave frequencies because it relies on coupled line theory, requiring small gaps between lines that are difficult to fabricate accurately at higher frequencies, thus limiting its practicality. On the other hand, alternative techniques, such as time delay, used in PMC packaged or Inverted Microstrip Line Gap Waveguides, share similarities with PRGW but demand additional dielectric layers, leading to increased costs and complexity. Despite efforts to improve performance with matching stubs, these implementations still exhibit significant phase imbalances over a limited bandwidth, typically around 16% [8]. Recognizing the limitations of single-layer network structures in terms of compactness and bandwidth, there has been a shift towards multi-layer configurations in beamforming networks. Multi-layer setups offer the advantage of compacting beamforming networks while minimizing phase imbalance [9]. Moreover, various alternative mechanisms based on Metallic Ridge Gap Waveguides, such as ferrite-based, dielectric-filled, and glide symmetrical holes [10,11,12], have been explored. However, these structures often suffer from issues like large size, limited bandwidth, and poor phase imbalance. Additionally, implementing a multi-layer configuration requires a high-tolerance fabrication process, which is currently not cost-effective.

These collective challenges underscore the urgent need for further research and innovation in millimeter-wave phase shifter design. Specifically, there is a critical demand for the development of compact, wideband phase shifters with minimal phase imbalances, particularly for application in multi-layer beamforming networks. Addressing these challenges will be paramount for advancing the capabilities of future wireless communication systems, including the anticipated evolution to 6G technology. Building upon previous work, this paper presents a comprehensive investigation of compact ultra-wideband printed ridge gap waveguide phase shifters for millimeter-wave applications. The main focus of this study is to overcome the limitations of single-layer technology by adopting a multi-layer coupling technique between two resonant patches. This approach offers a wider operating bandwidth and improved matching level and phase balance compared to previous designs. Additionally, this study introduces a novel analytical method for calculating the cutoff frequency and propagation constant of the PRGW structure, providing a new way to characterize PRGW structures that has not been addressed before. A systematic design procedure is employed to achieve a range of phase shift values from $0^{\circ }$ to $135^{\circ }$ over a broad frequency bandwidth centered at 30 GHz. To validate the effectiveness of the proposed design approach, a $45^{\circ }$ phase shifter is fabricated and experimentally evaluated. The measurement results demonstrate an impressive bandwidth of 10 GHz, covering frequencies from 25 GHz to 35 GHz, with a phase balance within ±$5^{\circ }$ throughout the operating bandwidth and a deep matching level of −25 dB. These findings highlight the superior characteristics of the proposed phase shifter, including its compactness, low loss, and low dispersion, positioning it as a promising candidate for millimeter-wave applications, particularly in B5G/6G wireless communications.

This paper is structured as follows: Section 2 presents a theoretical analysis of the PRGW structure, introducing a novel analytical approach to calculate the cutoff frequency and propagation constant, which is crucial for characterizing the PRGW structure. Section 3 focuses on the proposed PRGW phase shifter, detailing the theoretical analysis and design methodology, including the multi-layer PRGW phase shifter analysis, the proposed design, and the design methodology, along with simulation results. Section 4 presents the measurements and validation of the fabricated phase shifter, including comparisons with state-of-the-art designs to highlight its advantages. Finally, Section 5 concludes the paper by summarizing the findings and suggesting potential directions for future research.

## 2. Theoretical Analysis of PRGW Structure

In the field of electromagnetic engineering, while perfect electric conductors (PECs) are commonly found in nature, perfect magnetic conductors (PMCs) are engineered to exhibit properties resembling ideal magnetic surfaces within specific frequency bands [13,14,15]. These engineered PMC surfaces, also known as artificial magnetic conductors (AMCs) or band-gap surfaces, serve as essential components in various electromagnetic applications, facilitating precise control over wave propagation and manipulation of electromagnetic fields. One notable technology that exploits the capabilities of AMC surfaces is the Ridge Gap Waveguide (RGW) [16,17,18,19,20]. RGW configurations typically consist of two parallel plates, one featuring carefully designed periodic textures intended to control the propagation of electromagnetic waves. By purposefully arranging these periodic textured cells, RGW systems effectively confine electromagnetic energy within the waveguide structure, thereby preventing wave leakage and maintaining a quasi-TEM (transverse electromagnetic) mode within the air gap. This arrangement utilizes the unique properties of AMC surfaces, enabling RGW to establish a parallel-plate band-gap and facilitate precise control over electromagnetic waves in millimeter-wave and terahertz frequency regimes.

**Figure 1:**
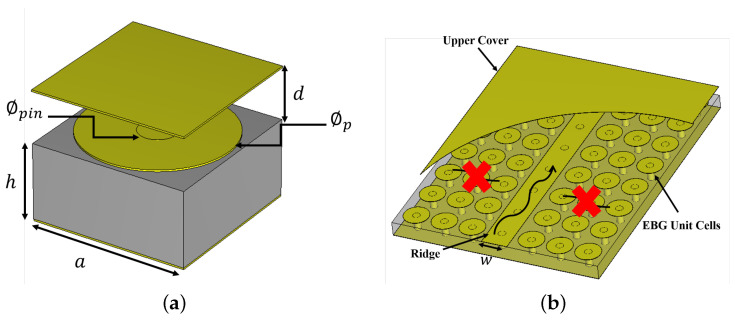
Geometrical configuration of the proposed PRGW structure, showing (**a**) the unit cell and (**b**) the PRGW, with dimensions *a* = 1.6 mm, *h* = 0.762 mm, $\phi_{pin}$ = 1.5 mm, *d* = 0.5 mm and *w* = 1.37 mm.

RGW technology encompasses various configurations, one of which is the Printed Ridge Gap Waveguide (PRGW). The development of PRGW represents a significant advancement in electromagnetic engineering, leveraging the principles of artificial magnetic conductors (AMCs) discussed previously. PRGW technology has received considerable attention in recent years, primarily due to its demonstrated low losses in the millimeter/terahertz-wave spectrum [21,22,23,24]. Unlike traditional waveguides, the PRGW structure shown in Figure 1 is constructed on printed circuit boards (PCBs), with the wave propagating over an air gap where dielectric losses are minimal. This remarkable design aspect significantly mitigates transmission loss, ensuring efficient signal propagation with minimal distortion and preserving signal integrity, crucial for high-frequency applications. Leveraging the quasi-TEM mode, which exhibits lower dispersion, minimizes degradation of signal quality, particularly important for maintaining signal transmission without distortion [21,22]. Its utilization in fields such as passive devices and antenna systems exemplifies the significance of microwave engineering in modern telecommunications [20,25,26,27].

The construction of the Printed Ridge Gap Waveguide (PRGW) involves a periodic arrangement of unit cells on a printed circuit board (PCB). Each unit cell, as shown in Figure 1a, consists of an artificial magnetic conductor (AMC) surface covered by a ground plane on the top. This configuration creates a band-gap where wave propagation is inhibited within a specific frequency range. However, by introducing a ridge between these periodic electromagnetic band-gap (EBG) cells, wave propagation is supported within the band-gap, enabling efficient signal transmission. The cutoff frequency is typically defined for the PRGW line rather than for the individual unit cells because the insertion of the ridge reduces the band-gap compared to the bandwidth of the band-gap of the periodic unit cells. Inserting the ridge disrupts the infinite periodicity assumed when calculating the band-gap bandwidth. Traditionally, the cutoff frequency and operating bandwidth of the PRGW are determined using eigenmode solvers in simulation tools. By appropriately defining the boundary conditions, the dispersion diagram can be extracted, and the bandwidth can be obtained along with the cutoff frequency value [28]. This conventional approach requires extensive use of simulation tools to calculate the initial dimensions needed to achieve the desired cutoff frequency and bandwidth. This process is often time-consuming and relies on a trial-and-error method, which lacks physical intuition and efficiency. In contrast, this work proposes an analytical procedure to calculate the cutoff frequency of PRGW. This method is based on the equivalent circuit model of the mushroom cell and its design procedure [29]. By employing this analytical approach, we aim to provide a more efficient and insightful method for determining the cutoff frequency and bandwidth, reducing dependency on simulation tools and expediting the design process.

**Figure 2:**
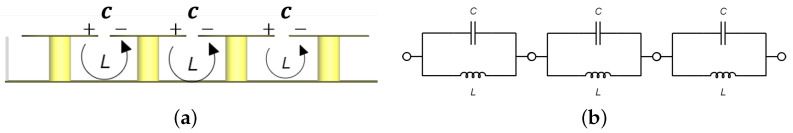
Geometrical configuration of the AMC surface and its equivalent circuit model. (**a**) Unit cell representing the structural elements, and (**b**) an equivalent circuit model showing the electrical counterparts.

Building on the previous discussion of the PRGW structure and its performance, the focus now shifts to the equivalent circuit model of the Electromagnetic Band-Gap (EBG) structure, particularly the mushroom-shaped unit cell depicted in Figure 1. Our analytical approach, which is essential for accurately predicting the behavior of PRGW systems, relies on this circuit model. The primary objective here is to determine the cutoff frequency of the PRGW analytically and to validate these results through comparison with simulated data. Traditionally, this determination is accomplished by plotting the dispersion diagram using CST and extracting the cutoff frequency via the eigenmode solver, as outlined earlier. To understand this process, consider that when an electromagnetic wave impinges on an array of mushroom-shaped unit cells, it induces electric fields across the gaps between the cells. These fields can be effectively modeled as an equivalent capacitance, denoted as *C*. Additionally, the incident fields generate currents that flow between adjacent unit cells, creating current paths through the walls or vias. This behavior can be represented by an equivalent inductance, *L*. Consequently, the equivalent circuit model of each unit cell comprises a parallel combination of capacitance *C* and inductance *L*, as shown in Figure 2 [29,30].

The surface impedance of such a structure can be calculated using the following equation:

(1) $$Z_{s}=\frac{j\omega L}{1-\omega^{2}LC}$$

where the resonance frequency is:

(2) $$\omega_{o}=\frac{1}{\sqrt{LC}}$$

The previously mentioned inductance and capacitance account for both the geometry of the individual unit cells and their overall geometric configuration. Using these parameters, the capacitance of each unit cell can be calculated as follows [29]:

(3) $$C=\frac{\phi_{p}\epsilon_{o}\left(\epsilon_{r}+1\right)}{\pi \sqrt{3}}\cosh{\left(\frac{a}{g}\right)}^{-1}$$

where $\epsilon_{o}$ and $\epsilon_{r}$ are the permittivities of the air and the substrate material, respectively, while $\phi_{p}$ is the diameter of the circular patch unit cell.

**Figure 3:**
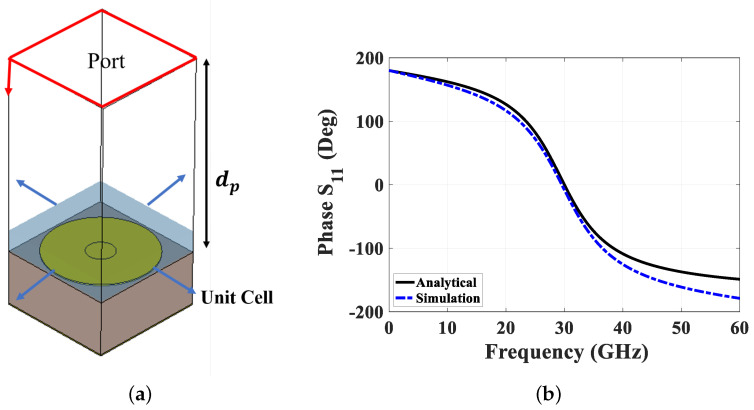
Phase reflection analysis simulation setup. (**a**) Simulated unit cell illustrating phase behavior, and (**b**) a comparison between analytical and simulated phase values.

From this capacitance, the inductance can be found using the following relation:

(4) $$L=\frac{1}{C\omega_{o}^{2}}$$

where $\omega_{o}$ is the angular resonance frequency, calculated as $\omega_{o}=2\pi f_{o}$, with $f_{o}$ being the center operating frequency, specified as 30 GHz in our work. With the values for the circuit elements determined, the surface impedance $Z_{s}$ can be calculated using Equation (1). The reflection phase of the proposed structure can be determined using the following equation [30]:

(5) $$∠S11=ℑm(\ln\left(\frac{\eta_{o}\left(\omega^{2}LC-1\right)+j\omega L}{\eta_{o}\left(1-\omega^{2}LC\right)+j\omega L}\right))$$

where $\eta_{o}$ is the characteristic impedance of air. From the reflection phase, the operating band of the proposed unit cell can be predicted, with the fractional bandwidth lying between $+90^{\circ }$ and $-90^{\circ }$. Thus, the cutoff frequency is the lower frequency $f_{l}$, corresponding to a phase of $90^{\circ }$. This analytical procedure is verified using CST-Microwave Studio, as shown in Figure 3a, where the unit cell is simulated as a periodic structure on two main axes. The reflection phase from this simulation is compared with the analytical results, as shown in Figure 3b, demonstrating good agreement between both methods.

The full characterization of the PRGW structure requires an examination of its wave propagation phase constant. Wave propagation within the PRGW is enabled by inserting a ridge between the unit cells, extending beyond the EBG structure, as depicted in Figure 1b. This structure allows the wave to propagate in the gap over the ridge, where the propagation constant β serves as the primary parameter governing the phase behavior of the structure. The propagation constant inside the PRGW can be calculated as follows:

(6) $$\beta =\sqrt{k^{2}-k_{c}^{2}}$$

where $k=\frac{2\pi \epsilon_{eff}}{\lambda }$ and $k_{c}=\frac{2\pi }{\lambda_{c}}$ are the wavenumber and the cutoff wavenumber of the structure, while $\epsilon_{eff}$ and $\lambda_{c}$ are the effective permittivity and the cutoff wavelength of the PRGW, respectively. This analytical approach allows for determining propagation constants without reliance on CST software or other simulation tools. Understanding these propagation constants is crucial for comprehending the behavior of electromagnetic waves within the PRGW structure. Such insights are invaluable in the design of microwave devices, particularly components like the phase shifter that will be discussed later, where precise control over phase behavior is critical for optimal performance.

It is worth mentioning that the effective permittivity is used instead of the dielectric constant of the PRGW substrate to account for the complex field distribution and interactions within the waveguide structure. The accurate calculation of the effective permittivity $\epsilon_{eff}$ is crucial for determining the wavenumber *k* and, subsequently, the propagation constant β. In previous studies, the effective permittivity has been determined for Inverted Microstrip Gap Waveguide (IMGW) structures [31]. These studies provide an empirical equation for $\epsilon_{eff}$ based on the specific geometry and material properties of IMGW. However, this empirical equation cannot be directly applied to PRGW due to distinct structural differences between the two waveguides. These differences include the presence of a ridge in PRGW, while IMGW features a microstrip line on a dielectric substrate.

To bridge the gap between the theoretical and practical calculations of effective permittivity, an adjustment to the empirical equation used for the Inverted Microstrip Gap Waveguide (IMGW) was required. This adjustment process involved deriving a correction factor through a comprehensive non-linear curve fitting procedure. Given that PRGW experiences a reduced dielectric influence compared to IMGW, the effective permittivity ($\epsilon_{eff}$) in PRGW is naturally lower. This crucial insight guided the modification of the empirical equation, ensuring it accurately reflects the effective permittivity for PRGW. The refinement process entailed extensive simulations and analytical comparisons, iteratively adjusting the empirical formula to achieve a close match with the observed data for PRGW. Through this methodical approach, a precise calculation method for $\epsilon_{eff}$ in PRGW was developed, which contributes significantly to the literature by providing a tailored solution for this specific waveguide structure. This improved methodology enhances the accuracy of phase constant calculations, essential for the design of microwave components such as phase shifters. The modified equation for the PRGW effective permittivity differs from that of IMGW, exhibiting an exponential decay behavior, and is expressed as follows:

(7) $$\begin{array}{cc} \epsilon_{PRGW_{eff}} & =1.137e^{-0.07068\epsilon_{r}}\;[\frac{\epsilon_{r}+1}{2}\left(1-0.0004\;{\left(\frac{2W}{d}\right)}^{2}\right)\\ & -\frac{\epsilon_{r}-1}{2}\left(1+0.2794\left(\frac{d}{2W}\right)+4.4735{\left(\frac{h}{W}\right)}^{-0.3105}\right)\\ & -0.0009\left(1+1.3382{\left(\frac{W}{h}\right)}^{1.2748}\right)] \end{array}$$

**Figure 4:**
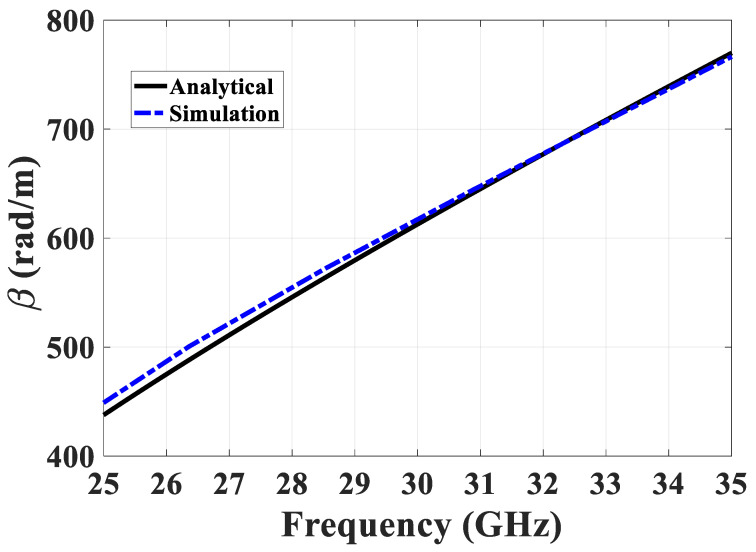
Comparison of analytical and simulated results for PRGW propagation constants, demonstrating the precision of the developed analytical model.

Following the refinement of the analytical approach and the derivation of $\epsilon_{eff}$ in PRGW, researchers can now accurately determine the propagation constant for the Printed Ridge Gap Waveguide (PRGW). Employing the analytical equation developed earlier, a comprehensive analysis is conducted for the PRGW structure, where the analytical predictions are compared to simulated propagation constants, as illustrated in Figure 4. This comparison reveals a high degree of agreement between the analytical and simulated results, affirming the accuracy of the analytical approach in predicting the propagation behavior of the PRGW structure.

## 3. Proposed PRGW Phase Shifter: Theoretical Analysis and Design Methodology

In this section, the application of Printed Ridge Gap Waveguide (PRGW) technology is explored for the development of a novel phase shifter. The design of the proposed phase shifter is founded on a directional multi-layer coupler architecture, utilizing the unique characteristics of PRGW. A comprehensive analysis of the coupling mechanism and the differential phase shift within the PRGW structure is undertaken. Following this analysis, the detailed geometry of the proposed design is presented, aimed at overcoming implementation challenges, such as achieving optimal matching and ensuring a flat phase shift response. In conclusion, we provide a concise summary of the design process employed for developing the phase shifter, highlighting key insights and considerations and presenting the simulated results to validate the effectiveness of the proposed design methodology.

### 3.1. Theoretical Analysis of Multi-Layer PRGW Phase Shifter

**Figure 5:**
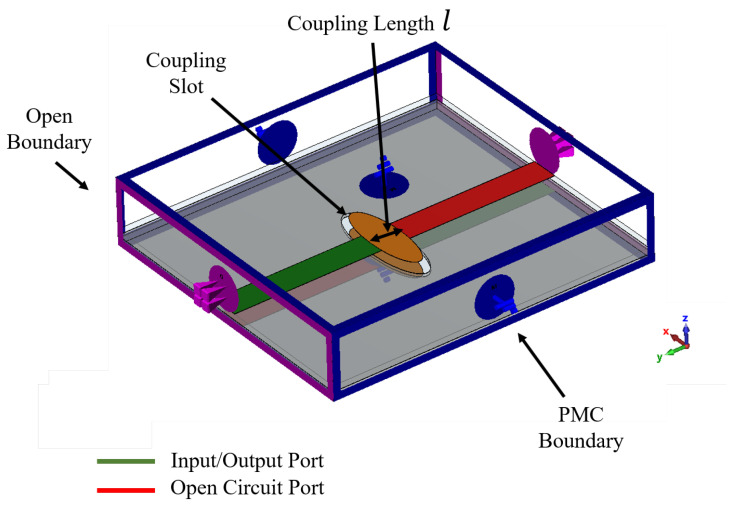
Ideal case for a multi-layer coupler with perfect magnetic conductor and open boundary conditions.

In the theoretical analysis of the proposed phase shifter, the fundamental design is described as a two-port device, derived from the modification of a multi-layer patch coupler shown in Figure 5 [32,33,34]. In this design, two ports are terminated with open circuits, while the remaining two serve as input and output ports. Critical performance indicators, such as the insertion loss and the return loss, are commonly used to evaluate microwave components. However, for a phase shifter, additional considerations are crucial. Among these are the differential phase shift’s accuracy and the phase response’s flatness. These specifications are essential to ensure that the phase shifter meets the rigorous standards of modern communication systems, particularly in scenarios requiring precise phase control.

To quantify the phase shift introduced by the proposed network, S-parameter network analysis is employed, assuming a coupling coefficient denoted as $C_{p}$. This phase shift is calculated relative to a PRGW reference line, which facilitates the assessment of phase changes caused by the proposed design. The phase shift obtained from the network is determined as follows: [32]:

(8) $$\phi_{c}=\frac{\pi }{2}-2\tan^{-1}\left(\frac{\sin\left(\beta l\right)}{\sqrt{1-{C_{p}}^{2}}\cos\left(\beta l\right)}\right)$$

In the previous equation, the used propagation constant β is obtained from the proposed Equation (6) and *l* is the physical length of the coupling structure, which is the secondary diameter of the elliptical shape, as shown in Figure 5. The phase shift obtained from the reference PRGW line is calculated as follows:

(9) $$\phi_{m}=-\beta l_{m}$$

Therefore, the differential phase shift can be calculated as follows:

(10) $$\Delta \phi_{c}=\frac{\pi }{2}-2\tan^{-1}\left(\frac{\sin\beta l}{\sqrt{1-C_{p}^{2}}\cos\beta l}\right)+\beta l_{m}$$

**Figure 6:**
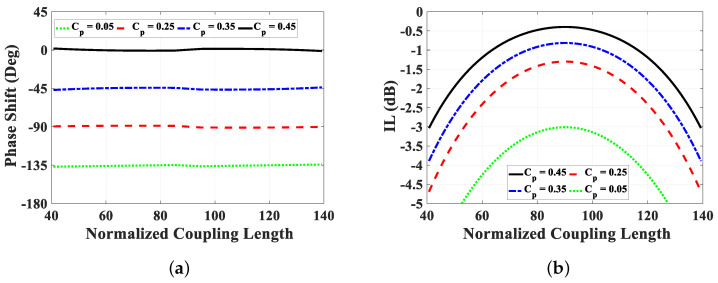
Phase shift and insertion loss variation due to changes in coupling parameters. (**a**) Phase shift, and (**b**) insertion loss.

Following the theoretical analysis, the achieved phase shift for various values of the normalized coupling length βl and the coupling coefficient $C_{p}$ are depicted in Figure 6. To achieve a wide range of phase shifts from $0^{\circ }$ to $135^{\circ }$, the physical length of the reference line $l_{m}$ is optimized to minimize the deviation in the differential phase shift. The estimated phase range extends from $0^{\circ }$ to $135^{\circ }$ for *C* values ranging from 0.45 down to 0.05. However, it is essential to evaluate the phase shifter’s operation by examining the return loss and insertion loss. As indicated by the equations in Table 1, the coupling value significantly affects the return loss $S_{11}$ and insertion loss $S_{21}$. This poses a limitation to our design in achieving high levels of phase shift. Specifically, for large phase shift values, the coupling value increases, leading to degradation in the return loss and, consequently, the insertion loss. Therefore, for large phase shift values, design modifications and tuning elements for matching are necessary, as will be discussed in the following section.

### 3.2. Proposed Multi-Layer PRGW Phase Shifter Design

**Figure 7:**
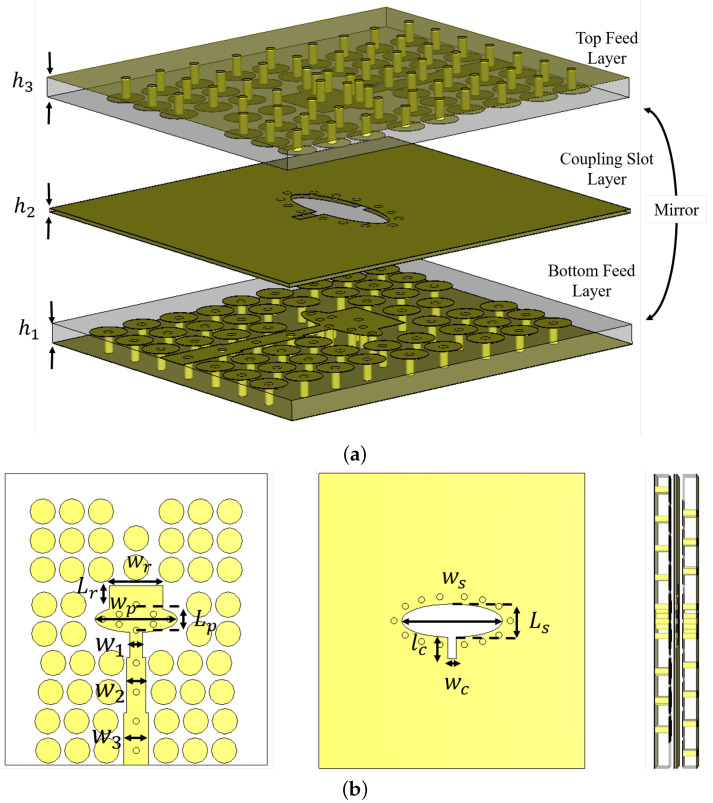
The geometry of the proposed PRGW phase shifter design. (**a**) 3D view, and (**b**) cross-sectional view.

The proposed configuration of the multi-layer PRGW phase shifter is illustrated in Figure 7a. This design primarily relies on a coupling section consisting of two elliptical patches positioned on the top and bottom layers. These patches are interconnected with the input and output PRGW lines. Proximity coupling between the patches is facilitated by cutting an elliptical slot in the ground planes of a thin RT6002 substrate with a thickness of $H_{2}$. Plated vias are placed around the coupling aperture to confine the electromagnetic field within the slot. The geometric parameters critical to the performance of the coupler include the width $W_{p}$ and length $L_{p}$ of the elliptical patches, as well as the width $W_{s}$ and length $L_{s}$ of the elliptical coupling slot. Additionally, a cut in the slot is included with dimensions $w_{c}$ and $l_{c}$ to provide another degree of freedom for fine control of the phase shift without affecting the matching level. These additional parameters are crucial for optimizing the phase shift independently of the matching level, ensuring robust performance of the phase shifter. All of these parameters are meticulously designed to ensure optimal coupling efficiency and minimal signal loss, thereby enhancing the overall performance of the phase shifter.

To address the theoretical analysis discussed previously, the design of the elliptical patches and slots ensures the desired coupling and differential phase shift. However, as indicated in the theoretical analysis, higher values of phase shift can degrade the return loss and insertion loss. To mitigate this, a matching transformer is incorporated into the proposed phase shifter, as shown in Figure 7b. This matching transformer consists of two lines with different widths to match the elliptical shape impedance to the 50 Ω line. In addition to the matching transformers, another modification to the multi-layer phase shifter is proposed to improve the matching level and add a degree of freedom to achieve the required phase shifter values while maintaining a deep matching level. A rectangular patch with dimensions $l_{r}$ and $w_{r}$ is added, and the elliptical slot is modified with a rectangular cut with dimensions $l_{c}$ and $w_{c}$, as shown in Figure 7b. These modifications help achieve phase shifters up to $135^{\circ }$, maintaining a deep matching level over the entire bandwidth, as will be demonstrated in the following section.

### 3.3. Design Methodology and Simulation Results

**Figure 8:**
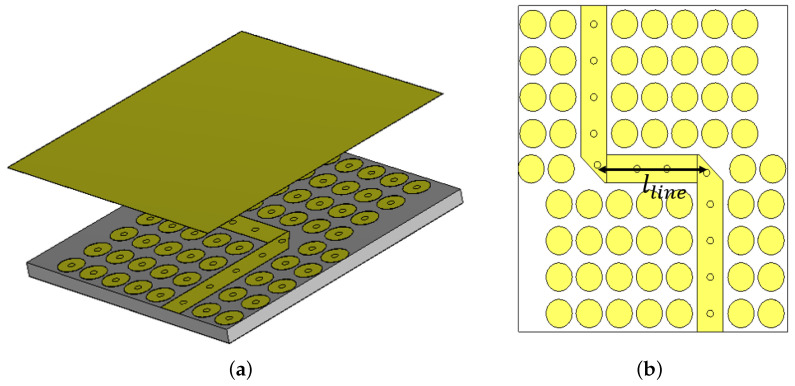
The geometry of the proposed PRGW reference line for phase comparison. (**a**) 3D view, and (**b**) cross-sectional view.

**Figure 9:**
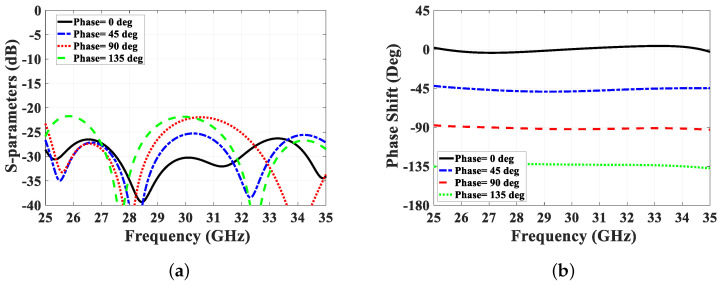
Phase shifter simulation results for different phase values. (**a**) S-parameter, and (**b**) differential phase.

The phase shifter is developed using a multi-layer coupler design, with two ports terminated as open circuits and the other two serving as input and output connections. The coupling mechanism from the bottom layer to the top layer follows the approach used in the multi-layer coupler proposed in [35]. The even and odd mode analysis conducted in that study is utilized to determine the initial dimensions for the phase shifter. The design methodology begins by selecting the required coupling value $C_{p}$ from the previously generated curves. These curves guide the design process for determining the values of $W_{p}$ and $W_{s}$ [35]. These values are subsequently optimized for each type of phase shifter, and the final tuned values are provided in Table 2. Moreover, the length of the elliptical patch $L_{p}$ is chosen to be equal to a quarter of the effective wavelength $\lambda_{eff}=\lambda /\sqrt{\epsilon_{eff}}$ at the center frequency of operation. Here, in our case, it is found to be around 1.5 mm, as depicted in Table 2. Once the necessary dimensions are established, the phase shift is compared to the reference line, as illustrated in Figure 8. This reference line is designed in a curved manner to ensure a flat phase response. The dimensions of the coupling slot and elliptical patch are fine-tuned along with other coupler parameters to achieve the desired performance. To achieve a good matching level and low insertion loss, particularly for low coupling values, a matching transformer is integrated into the coupling slot, as shown in Figure 7. Additionally, the length of the line $l_{line}$ is optimized to achieve the various phase shifts with a flat response. Simulation results are presented in Figure 9, where it is evident that the matching level for the proposed design is below 20 dB for all phase shift cases. Furthermore, the flatness of the phase shift curves is maintained within $\pm 5^{\circ }$ of the designed value ($0^{\circ }$ to $135^{\circ }$), demonstrating the effectiveness of the proposed design methodology. These simulation results underscore the capability of the proposed phase shifter to deliver accurate phase shifts with excellent matching and low insertion loss, fulfilling the stringent requirements of modern communication systems.

## 4. Measurements and Validation

**Figure 10:**
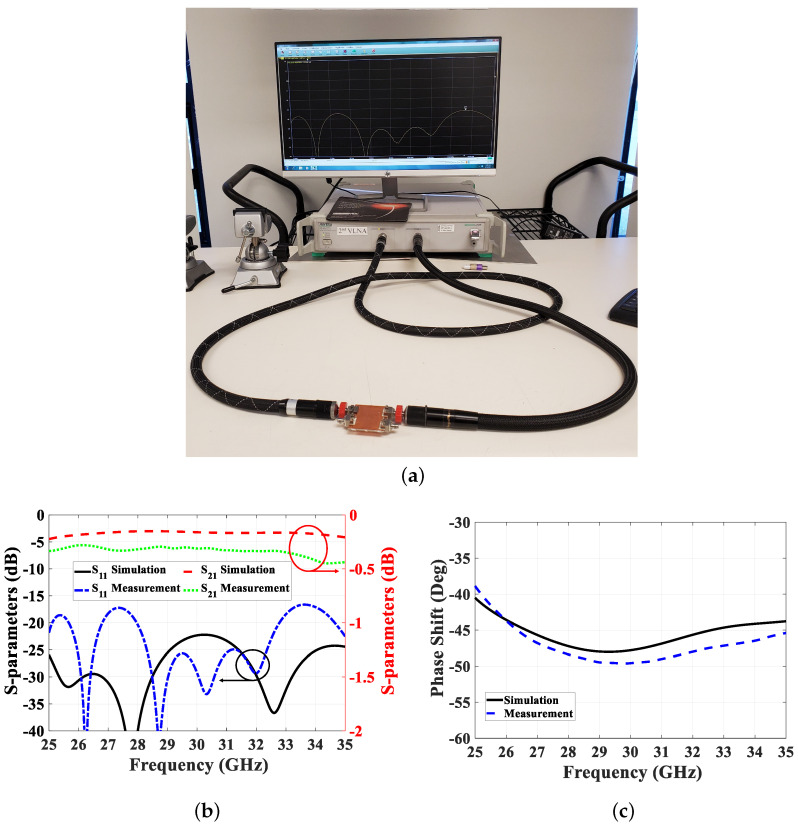
Phase shifter measurements verification with the simulation results. (**a**) Measurement setup, (**b**) S-parameter, and (**c**) differential phase.

The proposed PRGW phase shifter undergoes experimental validation through fabrication and measurement, as depicted in Figure 10a. We specifically select a $45^{\circ }$ phase shifter for validation, with detailed dimensions provided in Table 3. Fabrication involves assembling the PRGW phase shifter components using high-temperature and high-pressure epoxy. To ensure precise performance evaluation, we utilize a TRL calibration kit to mitigate any effects arising from connectors and microstrip line transitions. The S-parameters are measured using the ANRITSU MS46322A VNA, and a comparative analysis between the measured and simulated results is presented in Figure 10b. Notably, there is strong agreement between the measured and simulated S-parameters, achieving a relative bandwidth of 38% at 30 GHz. However, some discrepancies are observed in the measured results at the first of the band, primarily attributable to fabrication tolerances and the adhesive used during assembly. Furthermore, we evaluate the phase shift obtained from the phase shifter, comparing it with the reference line and simulated results, as depicted in Figure 10c. The proposed phase shifter demonstrates a phase balance of approximately $45\pm 5^{\circ }$ across the entire operational bandwidth. The proposed phase shifter not only demonstrates impressive phase balance but also exhibits desirable matching characteristics. The measured insertion loss remains below −0.5 dB, ensuring minimal signal attenuation. Additionally, the matching level achieved is below −16 dB, indicating efficient power transfer and minimal signal reflection within the operating bandwidth.

As we discuss the measurement and validation of our proposed multi-layer PRGW phase shifter, it is important to compare our work with the existing literature. While there is considerable research on phase shifters, particularly in millimeter-wave applications, the utilization of PRGW remains relatively limited. This highlights the unique contribution of our work, which seeks to address this under-explored area of research. By comparing our results with state-of-the-art methodologies documented in prior studies, we aim to underscore the advancements and distinctiveness of our PRGW-based phase shifter. The comparison Table 4 offers a thorough examination of the performance characteristics across various configurations of Ridge Gap Waveguide (RGW) phase shifters, providing insights into the existing literature in this field. Previous studies have primarily focused on Inverted Microstrip Gap Waveguide (IMGW) and Metallic Ridge Gap Waveguide (GGW) technologies, utilizing techniques such as Schiffman phase shifters [7] and time delay methods [8,9]. Schiffman phase shifters have been effectively utilized to construct linear Butler matrices and implement 1D beam scanning antenna arrays. However, these implementations face challenges due to their narrow operational bandwidth (20%) and large size [7]. These challenges are attributed to the reliance on coupled line theory, which necessitates small gaps between the coupled lines to achieve a wide range of phase shifts. Fabrication limitations make it difficult to achieve these small gaps while maintaining a reasonable matching level, particularly at millimeter-wave frequencies. Similarly, time delay techniques, including those used in PMC packaged or Inverted Microstrip Line Gap Waveguides, have been explored as alternatives to PRGW. However, these methods require additional dielectric layers, resulting in increased costs and complexity, while exhibiting significant phase imbalances over limited bandwidths (around 16%) [8,9]. In contrast, metallic RGW and metallic GGW technologies, while offering promising performance characteristics, face challenges such as high fabrication costs and bulky structures limiting their practical applicability [11,12,36,37]. Conversely, our investigation introduces a new perspective by exploring the application of PRGW in phase shifter designs. This change in focus is notable given the relatively limited exploration of PRGW-based approaches, despite their potential advantages. Unlike phase shifter designs utilizing IMGW and GGW technologies, which often encounter challenges, such as limited bandwidth and less-than-optimal phase balance, our PRGW-based approach offers distinct advantages. While some existing designs achieve better than $\pm 5^{\circ }$ degrees phase balance, typically around $\pm 4^{\circ }$, they often come with narrower bandwidth and larger size compared to our proposed design. Our approach using PRGW technology achieves a wider bandwidth and compact size while maintaining competitive phase balance performance. This trade-off is due to the optimized configuration of PRGW, which balances between phase performance, bandwidth, and size constraints. Specifically, our phase shifter designs leverage PRGW’s inherent benefits, including enhanced matching levels and phase stability. This focus on PRGW-based designs enables us to overcome many of the limitations associated with IMGW and GGW technologies, ultimately resulting in improved performance metrics. Furthermore, the proposed work emphasizes the significance of multi-layer configurations as a promising approach for compact beamforming networks. Notably, the prior literature has largely overlooked this aspect. Only a single previous study has investigated multi-layer phase shifters based on IMGW technology, with limited bandwidth and performance metrics [9]. In contrast, our innovative design methodology enables the realization of a broad range of phase shifts from $0^{\circ }$ to $135^{\circ }$, while ensuring a deep matching level across the entire spectrum. This capability represents a significant advancement over existing methodologies, which often struggle to achieve comparable levels of performance.

## 5. Conclusions

In this paper, we have introduced compact Printed Ridge Gap Waveguide (PRGW) phase shifters designed for millimeter-wave applications, focusing on achieving wide operating bandwidth, improved matching, and enhanced phase balance compared to single-layer technology. A unique methodology has been presented for achieving the required phase shift in PRGW technology, which has not been previously explored. Additionally, a novel analytical approach to calculate the cutoff frequency and propagation constant of the PRGW structure has been introduced, providing a new method to characterize PRGW structures. By employing multi-layer PRGW technology, we have enabled the realization of multi-layer beamforming networks without crossing, supporting wideband operation in a compact form factor. Our design procedure has successfully achieved phase shift values ranging from $0^{\circ }$ to $135^{\circ }$ over a broad frequency bandwidth centered at 30 GHz. A 45-degree phase shifter has been fabricated and measured, demonstrating a 10 GHz bandwidth (approximately 33% fractional bandwidth) from 25 GHz to 35 GHz, with a phase balance maintained within $45\pm 5^{\circ }$ and a deep matching level of −20 dB. These results underscore the superior performance of the proposed phase shifter in terms of bandwidth, phase balance, and matching levels, making it a promising candidate for advanced millimeter-wave applications, including beyond 5G (B5G) and 6G wireless communications. The proposed design, capable of implementing a wide range of phase shifts, is particularly advantageous for developing scalable beamforming networks such as 4 × 4 and 8 × 8 Butler matrices, accommodating the varying phase shift values required for larger networks.

## Figures and Tables

**Table 1:** S-parameters equations depending on the even-odd analysis of multi-layer coupler [32].

Quantity	Expression
$S_{11}$	$\frac{1-C_{p}\left(1+\sin^{2}\left(\beta l\right)\right)}{{\left[\sqrt{1-{C_{p}}^{2}}\cos\left(\beta l\right)+j\sin^{2}\left(\beta l\right)\right]}^{2}}$
$S_{21}$	$\frac{2jC\sqrt{1-{C_{p}}^{2}}\sin\left(\beta l\right)}{{\left[\sqrt{1-{C_{p}}^{2}}\cos\left(\beta l\right)+j\sin^{2}\left(\beta l\right)\right]}^{2}}$

**Table 2:** Phase shifter dimensions for different values of phase shift.

Parameter	$0^{\circ }$ Phase	$90^{\circ }$ Phase	$135^{\circ }$ Phase
$W_{p}$ (mm)	4.57	3.67	3.75
ine $L_{p}$ (mm)	1.48	1.55	1.5
$W_{s}$ (mm)	5.53	6.9	7
ine $L_{s}$ (mm)	1.8	1.53	1.1
$L_{line}$ (mm)	4.9	2.9	2

**Table 3:** $45^{\circ }$ phase shifter dimensions.

Parameter	Value (mm)	Parameter	Value (mm)
$h_{1}$	0.762	$l_{line}$	3.6
$h_{2}$	0.127	$W_{1}$	0.7
$W_{2}$	1.05	$W_{3}$	1.377
$W_{p}$	4.5	$L_{P}$	1.3
$W_{r}$	0.762	$L_{r}$	1.3
$W_{s}$	5.5	$L_{s}$	1.85
$W_{c}$	0.48	$L_{c}$	1.17

**Table 4:** Performance comparison between different RGW phase shifter configurations.

Ref.	RL [dB]	IL [dB]	$f_{o}$ (GHz)	BW %	Ph. Balance	Size ($\lambda_{o}^{2}$)	Tech.
[7]	14	1	30.71	20	$45\pm 3.5^{\circ }$	-	PRGW
[9]	-	-	30	16.7	$45\pm 3^{\circ }$	1×2	IMGW
[8]	-	0.6	29	13.8	$45\pm 3^{\circ }$	0.6×0.3	IMGW
[12]	10	0.7	26	26.3	$85\pm 5^{\circ }$	2.5×3.6	RGW
[11]	15	-	27	29.63	$90\pm 4^{\circ }$	2.9×4.6	RGW
[36]	25	-	94	6.4	$90\pm 4^{\circ }$	2.6×3.2	GGW
[37]	12	0.75	69.5	15.8	-	-	GGW
This Work	16	0.5	30	33.33	$45\pm 5^{\circ }$	1 × 1	PRGW

## Data Availability

Data are contained within the article.
